# Is one midwife per birthing mother an achievable goal? Education and leadership pathways for midwifery models of care in the Middle East

**DOI:** 10.3389/fgwh.2026.1795008

**Published:** 2026-04-08

**Authors:** Atika Khalaf

**Affiliations:** 1 The PRO-CARE Group, Faculty of Health Science, Kristianstad University, Kristianstad, Sweden; 2 Hind Bint Maktoum College of Nursing and Midwifery, Mohammed Bin Rashid University of Medicine and Health Sciences, Dubai Health, Dubai, United Arab Emirates

**Keywords:** Birthrate Plus®, Middle East, midwife-led continuity of care, one-to-one intrapartum support, safe staffing (NICE NG4), United Arab Emirates, WHO labour care guide, whole-time equivalent

## Abstract

Midwifery models of care (MoC), where midwives are the primary, continuous care providers across pregnancy, labor and the early postnatal period, are central to World Health Organization (WHO) guidance and are associated with fewer interventions and better maternal experiences compared to multi-professional teams. Yet in many Middle Eastern health systems, services remain obstetrician-led, midwifery education is uneven, and midwives’ professional autonomy is constrained. This mini-review synthesizes recent global guidance and regional evidence to outline education and leadership styles for accelerating a transition to midwifery MoC, with a pragmatic case example from the United Arab Emirates (UAE). I clarify what “one-to-one midwifery care” means in practice, summarize outcome evidence for midwife-led continuity and continuous intrapartum support, highlight regional progress and gaps in education, regulation and leadership, and provide a transparent, scenario-based staffing calculation for achieving one-to-one intrapartum care in the UAE using births-per-midwife planning ratios widely applied in service design. I conclude with a concise leadership and education roadmap, curricular alignment with the International Confederation of Midwives/WHO standards, protected leadership roles in governance, regulatory modernization for autonomy, and significant workforce expansion, arguing that one-to-one midwifery care is achievable and cost-sensible if implemented via phased, education-led reform.

## 1 What exactly is the “one-to-one midwifery model”?

In this review, the “one-to-one midwifery model” refers to continuous, one-to-one support provided by a registered midwife to a woman in established labor, delivered within a broader midwife-led continuity model that spans antenatal, intrapartum, and postnatal care, with timely referral and collaborative management when complications arise (1, 2). Crucially, one-to-one intrapartum support is a care standard within a midwifery MoC and should not be mixed with general workforce-density metrics (e.g., midwives per population or per 1,000 live births), which describe availability but do not, on their own, guarantee bedside one-to-one care. Three elements clarify this distinction. First, one-to-one intrapartum support is a standard of bedside care. Second, midwife-led continuity of care (MCoC) is the broader service model within which that standard is embedded. Third, workforce-density indicators (e.g., midwives per population) describe availability but do not ensure delivery of bedside one-to-one care.

This framing aligns with international guidance: WHO intrapartum recommendations, which emphasize respectful, continuous support and shared decision-making (1); the WHO Labour Care Guide operationalizes woman-centered, evidence-based intrapartum care (3); and the 2024 WHO's global position paper calls for countries to transition to midwifery MoC as a system strategy (2). The National Institute for Health Care Excellence in the UK (NICE) safe-staffing guidance specifies supportive one-to-one care in established labor (4); Birthrate Plus® (BR+) provides a planning methodology linking annual births to the required number of midwifery Whole-Time Equivalent (WTE) posts; that is, full-time staffing equivalents, to deliver that standard (4, 5). Table 1 presents the key workforce planning terms used in this paper.

**Table 1:** Key workforce planning terms used in this paper.

Term	Definition (as used in this paper)
Whole-time equivalent (WTE)	A staffing measure representing one full-time midwife post. Part-time staff are aggregated into full-time equivalents for workforce planning.
Births-per-WTE ratio	A planning metric that estimates how many annual births can be safely supported per one WTE midwife, accounting for intrapartum acuity, indirect care time, leave, supervision, and training.
Establishment	The total number of funded WTE midwife posts required to deliver a defined care standard (e.g., one-to-one intrapartum support).
Availability metric	A population-level indicator (e.g., midwives per 1,000 live births) describing workforce density; it does not guarantee bedside one-to-one care.
Acuity-based staffing	A staffing methodology that adjusts workforce requirements according to case-mix, labor complexity, indirect care demands, and required relief capacity.

## 2 Evidence snapshot—outcomes that matter

The case for one-to-one midwifery care rests on three outcome domains: mode of birth, maternal experience, and neonatal safety. First, recent evidence consistently shows that the MCoC improves key outcomes without signs of harm (6, 7). An updated Cochrane review (17 trials; 18,533 women) reported that, compared with other models, MCoC increases spontaneous vaginal birth (RR 1.05, 95% CI 1.03–1.07), reduces caesarean section (RR 0.91, 0.84–0.99) and instrumental birth (RR 0.89, 0.83–0.96), and may reduce episiotomy (7). The review further shows that, while women report more positive experiences, neonatal outcomes are similar across models, and no safety signal was identified (7). Second, continuous intrapartum support itself contributes independently to improved labor progress and reduced intervention. Continuous intrapartum support (from a midwife, doula, or chosen companion) is associated with shorter labors and lower analgesia use, with no evidence of harm (8, 9). Third, emerging evidence from Middle Eastern settings demonstrates that these benefits are transferable to regional contexts. A quasi-experimental study from Iran found that team-midwifery continuity significantly increased exclusive breastfeeding at 6 weeks postpartum (84%) compared with the control group (61%), improved 1- and 5-minute Apgar scores, and reduced neonatal intensive care unit (NICU) admissions (1% vs. 9%) (10). In a 2025 retrospective cohort study conducted in Iranian private hospitals, midwifery-led (private midwife) care was associated with significantly better labor pain control and lower rates of labor induction and episiotomy, while neonatal outcomes (including Apgar scores, need for resuscitation, breastfeeding initiation, NICU admission, and infant weight) were comparable to those under routine midwifery care (11).

## 3 Middle East status—education, regulation, and leadership

Three system-level levers determine whether one-to-one midwifery care can be implemented at scale: education, regulation, and leadership. Across the Middle East, pathways into midwifery demonstrably vary: some countries run direct-entry bachelor's degrees alongside “bridging” or post-nursing routes, and program standards/accreditation differ by institution and regulator. For example, Jordan's first national midwifery department (Jordan University of Science and Technology) offers both a 4-year direct-entry BSc in Midwifery and a 2-year BSc-bridging option for already-licensed practitioners, illustrating dual entry routes within a single system (12). In Saudi Arabia, universities such as Princess Nourah University and King Saud University offer bachelor-level midwifery programs aligned with national/international standards, while the Saudi Commission for Health Specialties also oversees a postgraduate midwifery curriculum pathway, indicating parallel academic and post-registration models (13, 14). In the United Arab Emirates, a direct-entry BSc in Midwifery is available at Fatima College of Health Sciences (with local accreditation details published), in line with a broader national strategy to strengthen nursing-midwifery education and regulation (15, 16). Lebanon likewise maintains direct-entry bachelor's training through institutions such as the Lebanese University and long-standing schools at USJ, reflecting country-specific educational traditions and frameworks (17, 18). Beyond program type, curricular alignment with the International Confederation of Midwives (ICM) competencies is uneven: a recent mapping study of Iran's bachelor-level midwifery curriculum against ICM's standards found areas rated only “relatively adequate” or “relatively inadequate,” underscoring variability in content and clinical exposure across the region (19). Region-wide syntheses from UNFPA further document heterogeneous midwifery education structures and accreditation practices across Arab States, reinforcing that there is no single, standardized pathway at present (20, 21).

However, a practical near-term agenda is to align all programs with the ICM's Global Standards, expand high-quality clinical placements and simulation, and invest in educator capacity alongside clear postgraduate pathways (e.g., advanced practice, education, leadership) to improve retention and capability (22). These priorities aligned with the ICM Standards (22) and are reinforced by regional workforce diagnostics according to the UNFPA's Arab States reports (20). Regional analyses demonstrate that several Arab states have already expanded direct-entry midwifery education and strengthened regulatory recognition of midwives as autonomous practitioners, though implementation varies (20, 21).

Regulatory arrangements also vary widely, and in several countries, midwives continue to practice under physician-dominated oversight, constraining their ability to lead physiological birth and deliver continuity models. Recent WHO guidance calls for transitioning to midwifery MoC backed by enabling regulation, clear scopes of practice, licensing pathways, and autonomous midwifery roles within integrated teams, while WHO's Eastern Mediterranean strategy highlights education, policy, and workforce management as linked powers for strengthening midwifery services (2, 23).

Moreover, leadership infrastructure is often thin: midwifery voices are under-represented in ministries, regulatory bodies, and provider governance, slowing the spread of midwife-led models. Establishing designated midwifery leadership roles and building leadership skills through structured development programs are repeatedly identified as accelerators for system-level change in ICM's Guide for Midwifery Leadership and recent leadership analyses. Embedding these roles in governance helps keep education reform, regulation, and service redesign moving in step (24, 25).

## 4 The case of the United Arab Emirates: is one-to-one midwifery care achievable?

To illustrate how these principles translate into operational planning, the UAE provides a pragmatic case example. The following “one-to-one midwifery MoC” treats bedside 1:1 intrapartum support as a care standard, not a headcount slogan.

### 4.1 UAE context

In the UAE, there were 101,088 registered live births in 2023 (26). Maternity care operates within a structurally mixed system, public and private, reflected in the federal/emirate regulatory system, the federal Ministry of Health and Prevention (MOHAP), alongside emirate regulators, such as the Department of Health - Abu Dhabi (DoH-AD), the Dubai Health Authority (DHA), and the Sharjah Health Authority, with each authority regulating services in its jurisdiction (27). These authorities regulate licensing, continuing professional development, and facility standards, and therefore provide potential mechanisms through which staffing benchmarks could be operationalized. Any shift toward acuity-based staffing would likely require coordinated guidance across federal and emirate regulators to ensure consistency in reporting and compliance frameworks.

The country has a robust nursing workforce; nurses and midwives together account for roughly 47% of the national health workforce (16). However, public datasets do not consistently disaggregate midwifery from nursing, echoing a broader challenge flagged by the State of the World's Midwifery 2021: even where workforce data exist, they are rarely fully disaggregated by occupation groups, which complicates precise midwifery headcounts and planning (28). The UAE health workforce is characterized by a high proportion of expatriate professionals, particularly in nursing and midwifery roles, reflecting broader national labor market patterns in the health sector (29, 30). This reliance on internationally recruited staff has implications for retention, workforce stability, and long-term domestic capacity building, particularly in light of Emiratization policies aimed at strengthening national participation in the health workforce (31, 32). National strategic documents indicate ongoing investment in nursing and midwifery workforce development, including the UAE National Strategy for Nursing and Midwifery (2022–2026), which prioritizes education expansion, Emiratization, scope-of-practice clarity, and leadership development (16). In addition, federal and emirate-level regulators have increasingly formalized licensing and continuing professional development requirements for maternity services (27). These policy directions provide structural conditions for assessing the feasibility of scaling midwifery-led MoC.

### 4.2 Translating births into midwifery staffing

In practical terms, achieving guaranteed one-to-one intrapartum care would require translating annual birth numbers into a funded midwifery establishment using an acuity-based methodology. At present, publicly available data do not indicate that standardized case-mix categorization tools are systematically embedded in UAE maternity staffing models (31, 33). To support consistent one-to-one care in established labor across settings and time periods, services could consider planning midwifery establishments using births-per-midwife ratios (measured in WTE) that account for intrapartum acuity, indirect care time, leave, supervision, and training requirements.

Two contrasting scenarios are presented in Table 2 to clarify the difference between availability benchmarks and true service-planning standards. The table presents two staffing scenarios for one-to-one intrapartum care in the UAE, based on 2023 births (101,088). Scenario A applies a BR + planning ratio (1 WTE per 29.5 births) to yield ≈3,430 WTE midwives, sufficient for 1:1 care with allowances for acuity and indirect time. Scenario B shows an advocacy availability metric [6 midwives per 1,000 births (34)], which translates to ≈607 midwives but does not assure bedside 1:1 support; it's a milestone, not a staffing model. Table 3 explains how to interpret the scenarios, and Supplementary Figure S1 illustrates a conceptual workforce flow for phased implementation of one-to-one intrapartum care.

**Table 2:** UAE staffing scenarios for achieving one-to-one intrapartum support^a^.

Parameter	Value/Assumption	Source/Notes
Registered live births (2023)	101,088	UAE national statistics
Scenario A ratio	1 Whole-Time Equivalent (WTE) per 29.5 births	Birthrate Plus® planning benchmark
Scenario A requirement	≈3,430 WTE midwives	Calculation: 101,088/29.5
Scenario B ratio	6 midwives per 1,000 births (availability metric)	WHO advocacy benchmark; not a staffing model
Scenario B headcount	≈607 midwives	Calculation: 101.1 × 6

a Table 3 below explains the interpretation.

**Table 3:** Interpretation of UAE staffing scenarios (based on 101,088 births).

Elements and interpretation
Scenario A—Birthrate Plus® benchmark for 1:1 care in established labor. Using a planning ratio of 1 WTE midwife per ∼29.5 births/year (typical BR+ range ≈ 28–30), the required establishment is ≈3,430 WTE midwives (101,088 ÷ 29.5). This level provides capacity for continuous one-to-one intrapartum support while accounting for indirect time, leave, supervision, and acuity.
Scenario B—“Midwives per 1,000 births” availability metric (advocacy benchmark). A commonly cited availability benchmark of ≥6 midwives per 1,000 live births yields a headcount of ≈607 midwives (101.1 × 6). Note: this is an availability metric; on its own, it does not guarantee bedside one-to-one support and is insufficient for safe labor-ward staffing. It can serve as a near-term milestone during workforce establishment and education scale-up.
Recommendation—Use Scenario A as the service-planning target for achieving one-to-one intrapartum care; track Scenario B only as an interim workforce milestone during scale-up.
Feasibility in the UAE—The UAE's sizeable nursing workforce, documented higher-education infrastructure, and national workforce strategy provide a foundation for phased midwifery expansion. Existing bachelor-level midwifery education (e.g., Fatima College of Health Sciences) and regulatory oversight across MOHAP, DOH-AD, and DHA indicate institutional capacity upon which bridging programs, direct-entry expansion, and postgraduate development could be built. While substantial scale-up would be required, these existing structures suggest that reform would build on, rather than create from scratch, national systems. Furthermore, while publicly available data on annual midwifery student intake and graduation numbers are limited, and interest in midwifery as a career among UAE nationals is not comprehensively reported, at least one accredited direct-entry program currently operates within the UAE higher education system (15). Expansion would therefore depend not only on institutional capacity but also on student recruitment strategies, career attractiveness, and alignment with national workforce localization priorities, supported by faculty development and strengthened clinical placement agreements.
Phasing proposal (illustrative)—To reach the Birthrate Plus® requirement of ≈3,430 WTE midwives, a phased 15–18-year horizon may represent a more operationally feasible course. Under a 15-year model, this would require an average net increase of approximately 180–220 WTE midwives per year; under an 18-year model, approximately 155–195 WTE per year. This extended timeline would allow incremental expansion of education capacity, stabilization of retention performance, and gradual redistribution of workforce roles without imposing excessive short-term strain on existing maternity and nursing services. For modelling purposes, we assume a 5% annual attrition rate under post-stabilization conditions, consistent with lower-bound turnover rates reported in international nursing workforce literature (39). Under a 15-year timeframe, aiming for a net increase of about 180–220 WTE midwives per year, a 5% annual attrition rate at full establishment (≈3,430 WTE) would mean around 170 midwives leaving each year. To achieve net growth, total annual recruitment might therefore need to be in the range of 350–390 midwives once the workforce approaches its target size. A simple sensitivity analysis illustrates the implications of higher turnover (39). If attrition were higher, around 8% (≈275 WTE) or 10% (≈343 WTE), annual recruitment needs could increase to approximately 455–495 or 525–565 midwives, respectively. These estimates illustrate how strongly workforce projections are influenced by retention patterns. Growth at this level would likely depend on gradually expanding local education capacity, including direct-entry programs and bridging pathways, as well as continued support for educators and clinical training sites. During the transition, temporary pressures may arise if experienced staff participate in training programs, potentially affecting workload distribution across some units. A staged approach, for example, early years focused on expanding education and strengthening retention, middle years consolidating growth and testing staffing adjustments, and later years moving closer to one-to-one targets, may help ease short-term pressures while maintaining maternity and emergency service coverage.

Operationally, integrating acuity weighting into the UAE system would not require a structural redesign of maternity services but rather the addition of standardised labour acuity scoring (e.g., complexity categories, induction rates, high-risk proportions, operative delivery rates) to routine service reporting. These data are already captured in hospital clinical records; the methodological shift would involve linking them transparently to WTE establishment calculations under regulatory oversight. Implementation would also carry funding implications, as increased WTE establishment requires sustained budget allocation across public and private sectors. Alignment between workforce commissioning and health financing structures would therefore be central to long-term sustainability.

Within the UAE's federal and emirate-level regulatory framework (MOHAP, DOH–AD, and DHA), a nationally coordinated technical standard may provide a useful mechanism to clarify these parameters and support maternity providers in evidencing compliance. At present, maternity services in the UAE are predominantly hospital-based and obstetric-led, delivered across public and private facilities. Public documentation does not indicate widespread implementation of formal acuity-based midwifery staffing tools such as BR+. Staffing models appear primarily structured around fixed shift allocations and service volume rather than explicit case-mix weighting. Dedicated freestanding midwife-led birth centers remain limited, and most births occur within consultant-led obstetric units or mixed-care environments (31). As an implementation benchmark, the UAE may adopt and localize the UK's BR+ methodology (35–37), used alongside the NICE NG4 safe staffing guidance (4), to translate clinical need into a transparent WTE requirement for labor wards and related maternity services (Table 3). This involves routine measurement of established labor hours, acuity categories, relief/break coverage, and escalation capacity, with results audited and reported to the relevant regulator. Therefore, staffing might be commissioned and inspected to the bedside standard (1:1 in established labor), using BR+ ratios and tools as the starting point, then calibrated to UAE case-mix, models of care, and service patterns. It is also noteworthy that the WHO's advocacy benchmark of ≥6 midwives per 1,000 live births is well below the midwifery staffing levels in most high-income countries, which range from 30 to 70 midwives per 1,000 live births (38).

### 4.3 Education and leadership roadmap

Implementation could be considered across three coordinated streams: education alignment, regulatory development, and leadership and governance strengthening. In the UAE context, an education and leadership roadmap might involve closer alignment of pre-service and continuing professional development with the ICM Global Standards, alongside the integration of competency-based curricula that reflect WHO's intrapartum recommendations and the WHO Labour Care Guide within teaching and assessment frameworks (1, 3, 22). This approach is intended to support graduates in developing the competencies required to provide respectful, evidence-informed, one-to-one intrapartum support and continuity of care. National clinical placement standards that facilitate exposure to community settings, midwife-led units, and obstetric units, alongside investment in educator capacity and simulation, could also be considered to further enhance clinical preparedness and workforce sustainability (22).

It is recommended that the UAE fund 12–18-month bridging programs enabling experienced labor-and-delivery nurses to qualify as registered midwives. In parallel, the UAE might consider expanding direct-entry midwifery programs and establishing clear postgraduate pathways (e.g., advanced practice, educator, and researcher roles) to support career development, retention, and leadership in the midwifery workforce (20). In addition, governance is recommended to begin by appointing Chief Midwife roles at national, emirate, and provider levels, with clear authority over staffing models, education commissioning, and quality standards. Regulations could then be updated to recognize the full scope of midwifery practice, including protocol-based prescribing of medicines (where appropriate) and independent caseload holding within integrated teams. In addition, all maternity providers would be required to undertake BR+ style reviews to set transparent WTE staffing levels for delivering one-to-one care in established labor, in line with NICE NG4 and NHS England's National Quality Board guidance on safe, sustainable staffing (4, 24). Moreover, accountability and incentives can be embedded by writing key indicators directly into service contracts, for example, the proportion of women receiving named-midwife continuity, the percentage who receive one-to-one support in established labor, and a respectful-care indicator, and then linking both payments and accreditation (for public and private providers) to independently verified performance on these measures. Regional analyses from UNFPA underscore that such combined investments in education, regulation and leadership are central to scaling midwifery models of care across Arab states (2, 20).

### 4.4 Limitations and research gaps

This analysis has several limitations. It relies on secondary, aggregate data rather than facility-level midwifery rosters or time-and-motion studies, and current UAE workforce statistics do not consistently distinguish nurses from midwives, limiting the precision of headcounts and deployment estimates. The BR+ ratio is induced from UK services and applied as an indicative benchmark, not yet calibrated to UAE-specific acuity, case-mix, or MoC, and the financial and educational capacity implications of scaling to ≈3,430 WTE midwives are not fully modelled. In addition, there is little empirical evidence on how to operationalize international standards (ICM, WHO, NICE, National Quality Board) within the UAE's federal/emirate mixed public-private system, including the design of enforceable BR+ style reviews and incentive frameworks. More broadly, robust regional data on midwife-led continuity models, cost-effectiveness, and women's experiences in Middle Eastern settings are scarce, underscoring the need for UAE-based observational, economic, and mixed-methods studies to test, refine, and sustain one-to-one midwifery care. Consequently, the staffing scenarios and implementation pathway proposed in this paper should be interpreted as a strategic framework for discussion rather than a definitive workforce prescription. In particular, attrition rates specific to UAE midwifery are not publicly disaggregated, and projections rely on internationally reported turnover ranges; future national workforce monitoring would allow more precise modelling. In addition, publicly available sources do not clearly indicate that formal acuity-based midwifery staffing systems are routinely implemented across maternity facilities in the UAE. Accordingly, the proposed BR+ -style modelling is presented as a potential framework for future development rather than as a reflection of current operational practice. Also, more detailed reporting of midwifery education intake and graduation rates in the UAE would allow more precise modelling of scale-up timelines. Finally, the short-term operational impact of workforce redistribution during scale-up is not modelled and would require detailed service-level workforce simulation.

## 5 Conclusion

One-to-one intrapartum support should be understood as a bedside care standard embedded within a broader MCoC model, and the international evidence base suggests associated improvements in maternal outcomes without signals of harm. However, the present analysis is conceptual and relies on secondary aggregate data and externally derived planning benchmarks. The workforce estimates presented are therefore indicative rather than predictive and should be interpreted as scenario-based illustrations rather than precise national requirements.

Across the Middle East, variation in education pathways, regulatory frameworks, and leadership infrastructure may influence the pace at which midwifery MoC are adopted. Greater alignment of curricula with ICM standards, clarification of the scope of practice and licensing arrangements, and the development of formal midwifery leadership roles within governance structures could support further advancement in this area. The UAE case illustrates that workforce modelling for one-to-one intrapartum care can be technically constructed within the national context; however, its practical feasibility would depend on concurrent investment in education capacity, retention strategies, regulatory coordination, and governance infrastructure. Importantly, this modelling is situated within documented national workforce strategies, existing education programs, and established regulatory authorities, which collectively provide an institutional foundation for reform. Nonetheless, empirical testing, financial modelling, and phased evaluation would be required before large-scale implementation. A phased scale-up, potentially combining bridging pathways, expansion of direct-entry programs, international recruitment, and investment in educator and leadership capacity, may offer a reasonable route toward strengthening midwifery services, contingent on retention performance and workforce stability. However, the pace, cost, and system readiness for such reform remain to be empirically evaluated within the UAE's mixed public-private regulatory environment.

## References

[1] WHO. Who Recommendations: Intrapartum Care for a Positive Childbirth Experience. Geneva: World Health Organization (2018); Copyright©. 30070803

[2] WHO. Transitioning to Midwifery Models of Care: Global Position Paper. Geneva: World Health Organization (2024).

[3] WHO. WHO Labour Care Guide: User’s Manual. Geneva: World Health Organization (2021).

[4] NICE. Safe Midwifery Staffing for Maternity Settings. UK: National Institute for Health Care Excellence (2015).

[5] Ball J Washbrook M. RCM. Working with Birthrate Plus®: How This Midwifery Workforce Planning Tool Can Give You Assurance About Quality and Safety. London: The Royal College of Midwives (2010).

[6] Kuipers YJ. The future of midwife-led continuity of care: call for a dialogue. Dialogues Health. (2024) 4:100170. 10.1016/j.dialog.2024.100170 38516226 PMC10953852

[7] Sandall J Turienzo CF Devane D Soltani H Gillespie P Gates S Midwife continuity of care models versus other models of care for childbearing women. Cochrane Database Syst Rev. (2024) 4(4):CD004667. 10.1002/14651858.CD004667.pub6 38597126 PMC11005019

[8] Bohren MA Hofmeyr GJ Sakala C Fukuzawa RK Cuthbert A. Continuous support for women during childbirth. Cochrane Database Syst Rev. (2017) 27(4):193. 10.1002/14651858.CD003766.pub6 PMC6483123 28681500

[9] Stjernholm YV Charvalho PDS Bergdahl O Vladic T Petersson M. Continuous support promotes obstetric labor progress and vaginal delivery in primiparous women–a randomized controlled study. Front Psychol. (2021) 12:582823. 10.3389/fpsyg.2021.582823 33679510 PMC7928421

[10] Shahinfar S Abedi P Najafian M Abbaspoor Z Mohammadi E Alianmoghaddam N Effect of continuity of team midwifery care on maternal and neonatal outcomes: a quasi-experimental study in Iran. Sci Rep. (2024) 14(1):22819. 10.1038/s41598-024-73751-8 39354021 PMC11445556

[11] Shahbazi Sighaldeh S Eskandari E Khosravi S Ebrahimi E Haghani S Shateranni F. Comparison of maternal and neonatal outcomes of midwifery-led care with routine midwifery care: a retrospective cohort study. BMC Nurs. (2025) 24(1):158. 10.1186/s12912-025-02789-4 39934823 PMC11817875

[12] JUST. Welcome: Jordan University of Science and Technology (2025). Available online at: https://www.just.edu.jo/FacultiesandDepartments/FacultyofNursing/Departments/Midwifery/Pages/Welcome.aspx (Accessed January 18, 2026).

[13] PNU. Bachelor of Science in Midwifery: Princess Nourah bint Abdulrahman University (2025). Available online at: https://pnu.edu.sa/en/Faculties/Pages/ProgramDetails.aspx?ProgramCode=NR-BS-MIDW&utm_source (Accessed January 18, 2026).

[14] KSU. About the Bachelor of Science in Midwifery Program: King Saud University (2024). Available online at: https://nursing.ksu.edu.sa/en/node/5064?utm_source (Accessed January 20, 2026).

[15] FCHS. Bachelor of Science in Midwifery Program: Fatima College of Health Sciences (2025). Available online at: https://www.fchs.ac.ae/midwifery-2/ (Accessed January 20, 2026).

[16] MOHAP. UAE National Strategy for Nursing/Midwifery: A Roadmap to 2026: Ministry of Health & Prevention. United Arab Emirates (2022). p. 50.

[17] Lebanese University. Faculty of Public Health: Lebanese University (n.d.). Available online at: https://www.ul.edu.lb/en/colleges-faculties-details/300/Faculty-of-Public-Health (Accessed January 18, 2026).

[18] Saint Joseph University of Beirut. School of Midwifery: Saint Joseph University of Beirut (2026). Available online at: https://usj.edu.lb/esf/ (Accessed January 18, 2026).

[19] Abdolalipour S Mohammad-Alizadeh-Charandabi S Babaey F Allahqoli L Ghaffari R Mirghafourvand M. Mapping of Iranian midwifery curriculum according to the international confederation of midwives competencies. BMC Med Educ. (2023) 23(1):791. 10.1186/s12909-023-04755-7 37875917 PMC10599037

[20] UNFPA. The state of the midwifery workforce in the Arab region (2022). Available online at: https://arabstates.unfpa.org/en/publications/state-midwifery-workforce-arab-region (Accessed January 15, 2026).

[21] Safari K McKenna L Davis J. Midwifery in Middle Eastern and north African countries: a scoping review. Women Birth. (2021) 34(6):503–13. 10.1016/j.wombi.2020.11.002 33199188

[22] ICM. ICM global standards for midwifery education (revised 2021). International Confederation of Midwives (2021).

[23] WHO. Strategy and policy: World Health Organization - Eastern Mediterranean region (2025). Available online at: https://www.emro.who.int/nursing/strategy/ (Accessed November 8, 2025).

[24] ICM. Guide midwifery leadership (2022).

[25] RCM. Leadership Project: Developing Midwifery Leadership Pathways in Practice and Education. London: Royal College of Midwives (2024).

[26] MOHAP. UAE statistical annual report 2023: United Arab Emirates Ministry of Health and Prevention (2025). Available online at: https://mohap.gov.ae/en/w/uae-statistical-annual-report-2023 (Accessed January 21, 2026).

[27] UAEGP. Health regulatory authorities: United Arab Emirates Government Platform (2025). Available online at: https://u.ae/en/information-and-services/health-and-fitness/health-authorities (Accessed January 21, 2026).

[28] UNFPA, ICM, WHO. The state of the world’s midwifery 2021: World Health Organization (2021). Available online at: https://www.who.int/publications/i/item/sowmy_2021 (Accessed January 21, 2026).

[29] Al-Yateem N Almarzouqi A Dias J Saifan A Timmins F. Nursing in the United Arab Emirates: current challenges and opportunities. J Nurs Manag. (2021) 29(2):109–12. 10.1111/jonm.12984 32100891

[30] Alshamsi AI. A review of the United Arab Emirates healthcare systems on medical tourism and accreditation. Front Health Serv. (2024) 4:1329252. 10.3389/frhs.2024.1329252 38449575 PMC10915079

[31] Koornneef E. Improving compliance with healthcare regulatory requirements in the United Arab Emirates (2019).

[32] Paulo MS Loney T Lapão LV. How do we strengthen the health workforce in a rapidly developing high-income country? A case study of Abu Dhabi’s health system in the United Arab Emirates. Hum Resour Health. (2019) 17(1):9. 10.1186/s12960-019-0345-9 30678690 PMC6346501

[33] El Azzabi S Salem EO. Midwifery care in the UAE. In: Al-Shamsi HO, editor. Healthcare in the United Arab Emirates. Singapore: Springer (2025). p. 351–61.

[34] WHO. Who calls for global expansion of midwifery models of care: World Health Organization (2025). Available online at: https://www.who.int/news/item/18-06-2025-who-calls-for-global-expansion-of-midwifery-models-of-care (Accessed January 23, 2026).

[35] Griffiths P Turner L Lown J Sanders J. Evidence on the use of birthrate plus® to guide safe staffing in maternity services–a systematic scoping review. Women Birth. (2024) 37(2):317–24. 10.1016/j.wombi.2023.11.003 38007331

[36] Cronie DJ Rosman A de Vries R. Measure to improve: a pilot study of birthrate plus in the Netherlands. Br J Midwifery. (2024) 32(6):302–8. 10.12968/bjom.2024.32.6.302

[37] He H Lu H Wang Y Pang R Qiu L Yao J Application of the birthrate plus (BR+) methodology to identify midwifery workforce shortages in China: a retrospective multisite study. Interdiscip Nurs Res. (2023) 2(2):100–6. 10.1097/NR9.0000000000000020

[38] Combellick JL Telfer ML Ibrahim BB Novick G Morelli EM James-Conterelli S Midwifery care during labor and birth in the United States. Am J Obstet Gynecol. (2023) 228(5):S983–93. 10.1016/j.ajog.2022.09.044 37164503

[39] Singh J Mohamed SGEA Mishra V Rana S. Unlocking retention: a prescriptive framework for retaining trained staff in critical care units. J Health Organ Manag. (2024) 38(8):1204–27. 10.1108/JHOM-04-2024-0142 40934214

