# Supplementary material for: Is one midwife per birthing mother an achievable goal? Education and leadership pathways for midwifery models of care in the Middle East
Source: Front Glob Womens Health. 2026 Apr 8;7:1795008. doi: 10.3389/fgwh.2026.1795008 (PMC13101474; doi:10.3389/fgwh.2026.1795008)
Supplement: Supplementary file 1 [file Datasheet1.pdf]

Supplementary Material

ANNUAL LIVE BIRTHS
101,088

⬇

CARE DEMAND (ACUITY-ADJUSTED)
Births-per-WTE ratio (1 WTE per 29.5 births)

⬇

WORKFORCE REQUIREMENT
Total midwives needed ≈ 3,430 WTE

⬇

WORKFORCE OUTFLOW
Annual attrition (5% steady state ≈ 170 WTE)

⬇

PLANNED WORKFORCE GROWTH
Net increase target: 180-220 WTE/year (15-year horizon)

⬇

ANNUAL RECRUITMENT NEED
Attrition replacement + growth ≈ 350-390 midwives/year

⬇

SUPPLY PIPELINE & LEVERS
• Direct-entry education programmes
• Bridging and upskilling pathways
• International recruitment
• Retention and workforce sustainability strategies

Figure 1. Illustrated Workforce Flow Model for One-to-One Midwifery Staffing in the United Arab Emirates. WTE = Whole Time Equivalent (full-time staffing equivalents).

Dubai Health - Internal Use

Figure 1 shows a conceptual workforce flow for phased implementation of one-to-one intrapartum care, where annual births are translated into required Whole-Time Equivalent (WTE) midwifery establishment using an acuity-adjusted planning ratio. Annual attrition is then subtracted, and gross recruitment requirements are calculated by combining attrition replacement with planned net workforce growth. Education pipeline capacity and retention performance determine the operational feasibility of projected intake targets.
